# Subjective social status among young working women in Sweden: how is it established and how does it affect health and well-being? A qualitative interview study

**DOI:** 10.1186/s12889-025-23645-9

**Published:** 2025-07-17

**Authors:** Ylva Lindberg, Åsa Andersén, Anna Nyberg, Ulrika Winblad, Ingrid Demmelmaier

**Affiliations:** Department of Public Health and Caring Sciences, Uppsala University, Box 564, Uppsala, 751 22 Sweden

**Keywords:** Thematic analysis, Mental health, Public health, Social media

## Abstract

**Background:**

Mental ill-health has increased among young people in Sweden in recent years, particularly among young women. One contributing factor could be a striving for high social status, communicated around the clock on social media platforms. The aim of the study was to explore how young working women in Sweden perceive social status and how it relates to their own health and well-being.

**Methods:**

The study sample included 15 women aged 25–35 years, recruited from the Swedish Longitudinal Occupational Survey of Health, a cohort derived from a representative sample of the Swedish working population. Maximum variation purposeful sampling was employed to achieve rich information in the data. Data were collected through individual semi-structured interviews and analysed using thematic analysis.

**Results:**

The analysis identified two main themes. The first theme, the *qualities and processes of subjective social status*, consisted of the subthemes *material and personal resources* and *interpersonal and contextual interplay*, and described aspects affecting social status, such as income, gender, and social life, as well as processes such as external influence and comparison. The second theme, *the influence of subjective social status on health and well-being* consisted of the subthemes *being judged by oneself and others* and *strategies to reduce negative impact*, and encompassed emotional responses to social status and approaches and attitudes to regulate its impact.

**Conclusions:**

The results indicate that subjective social status in young working women today is established through dynamic and interactive processes involving several important aspects beyond traditional socioeconomic measures. The findings regarding the impact of gender and foreign background on subjective social status suggest a need for continued efforts to strengthen equality and integration in Swedish society. The impact of processes around establishing and displaying subjective social status on well-being and health appears to vary, depending on each individual's approach and active management of, e.g., social media.

## Introduction

Mental ill-health, in the form of symptoms of common mental disorders (CMD) such as depression, anxiety, and stress-related disorders has increased among young adults in Sweden during the last 15–20 years [1, 2]. This development is particularly evident among young women [1, 2]. Mental ill-health is the most common cause of sickness absence in Sweden, especially for younger age groups [3]. The reasons behind increased mental ill-health and sickness absence in young people, particularly young women, are not fully understood. One factor that has been well-established as influencing both mental and physical health outcomes is an individual’s social status in society [4].

Social status can be defined as “the prestige, respect, and esteem that a party has in the eyes of others” ([5], p. 74), and as “an index of the social worth that observers ascribe to an individual or a group” ([5], p. 74). This definition indicates that social status of an individual or a group depends on the observer, not only on the individual or group; status is decided through the observers’ subjective evaluative processes. In different contexts and among different individuals, varying perspectives can guide these evaluative processes, meaning that different factors or combinations of factors can decide an individual’s status depending on the circumstances [5].

Social status has often been measured with one or more of three measures: years of education, occupational level (or occupational prestige), and income/wealth; measures which can be referred to as *objective social status* [6]. Another way to conceptualise social status is via *subjective social status* (SSS), in which individuals place themselves in a social hierarchy. This is often measured using a ladder scale where respondents indicate their position on the ladder compared to others in a given group. This group can refer to, for example, the entire country, or one’s community, based on education, income, and occupation [6]. The cognitive averaging principle states that an individual uses some average of different relevant factors to decide on their SSS. The averaging can also refer to different time points taken into consideration, i.e. an averaging across past, present, and expected social status [7–9].

### Determinants and outcomes of subjective social status

Determinants of SSS in different groups, such as the Swedish working population, American young adults, and British civil service employees, have been the focus of inquiry in previous quantitative studies. In addition to education, occupation, and personal income, SSS has been found to be connected to other factors; for example, household financial situation [8–10], job control, general life satisfaction [10], and satisfaction with standard of living [9]. A recent Swedish study indicated that objective measures were important determinants of social status, but that there were also other factors of importance; for instance, manners, appearance, residential area, and social network [11].

SSS has been found to be connected to both physical [9, 12–15] and mental health outcomes [9, 12, 14–19] and to explain variations in outcomes beyond more traditional objective measures. For example, SSS is associated with self-rated health [10, 12, 15, 20], musculoskeletal symptoms [10], allostatic load [21], sleep disturbances [10, 22], emotional exhaustion [10], depressive symptoms [10, 21], and psychological functioning [15, 22], even when controlling for the traditional measures of social status. Most studies investigating SSS have used large samples and quantitative analytic methods. Such studies are useful to identify comprehensive patterns in the data and produce results that can be generalised to larger populations. However, to gain a deeper understanding of individuals´ perceptions and experiences of SSS and its impact on well-being, qualitative methods have been applied. Research focusing on SSS utilising qualitative methods and interview data has focused on various groups, such as immigrants [23, 24], residents in assisted living [25], and adolescents [26–29]. Traditional socioeconomic measures have been described as important when rating SSS [23, 24, 27–29]. However, other determinants are also considered, such as strong values (e.g., kindness) [28, 29], language proficiency [23], health [25, 29], and social skills [25].

While results in the above studies centre on determinants of SSS, some of the studies (the ones focusing on adolescents) explored experiences of social status differences [28], strategies for positioning [27], and relation to illness [26] more broadly. Findings from these studies indicate that context matters—such as school culture [28]—and that different expectations for girls and boys were evident [26, 27]. Striving to reach the ideals according to the norm systems led to stress and psychological and somatic problems, and was more common among girls [26]. Both negative feelings, e.g. anger, jealousy, shame and anxiety, and positive feelings, e.g. optimism, motivation, and thankfulness have been found to result from comparison to others, as have negative and positive coping mechanisms [28].

The awareness of social status and its impact seems to be strong during adolescence. However, there is limited understanding of how these dynamics play out in other life stages. One group of special interest is young working women. Young women have more difficulty when trying to establish themselves in higher positions in the labour market [30], and are exposed to poorer psychosocial work environments [31], while also having the double burden of unpaid work [32], as well as being constrained by a narrower time frame than men regarding family formation. Therefore, young women are likely to struggle while establishing themselves in society.

### Social comparison and health

Central to the individual’s own appraisal of SSS is their comparison to others when placing themselves in the status hierarchy [6, 9]. Social comparison involves evaluating one’s opinions and abilities by comparing them to others’ opinions and abilities. If the comparison is unfavourable, the individual can feel inadequate and experience failure [33]. Comparing oneself to those who are worse off can lead to either the evaluation that the one comparing has a relatively advantaged position or that one’s own status could decline. Likewise, an upward comparison can result in the perception that one’s position is disadvantaged or that there is a possibility for improvement [34].

The process of social comparison can be present in all areas of life, such as in workplaces, family life, leisure activities, etc., and nowadays it is often present on social media platforms [35, 36], where the potential number of others to compare to is also far greater. Studies have found that unfavourable comparison on social media has a negative effect on psychological well-being [37] and mental health [38]. A recent research review however reported inconclusive results [39]; while some studies report associations between social comparison in social media and poor psychological well-being, others indicate positive associations with good mental health and a reversed association. A phenomenological study with young adult women in the U.S [40]. showed that online comparison could lead to jealousy or sadness and that exposure to social media content in general made participants question their personal and social status. When the participants did not find images to be realistic or failed to identify with them, it counteracted negative feelings.

In summary, mental ill-health and related sickness absence has increased among young women in Sweden in recent years, but the reasons are not entirely understood. Previous studies have shown that SSS is connected to mental health outcomes and that processes linked to social comparison can be both positive and negative for mental health. Comparing oneself with others and striving for high social status may be a key factor when it comes to experiences of stress and mental ill-health. However, it is not clear how social status is perceived among young working women in Sweden today, nor is it understood how young women perceive social status to affect them.

## Aim

The aim of the study was to explore how young working women in Sweden perceive social status and how it relates to their own health and well-being.

## Methods

### Design

An explorative design with semi-structured interviews and thematic analysis.

### Sampling technique and study population

Purposeful sampling with a maximum variation approach was used [41, 42]. Potential participants were identified among women aged 20–35 years—a formative age where one usually establishes oneself in working- and family life—who had answered the Swedish Longitudinal Occupational Survey of Health (SLOSH) questionnaire for persons who work in 2020, and who had indicated consent to be contacted for additional questions. A variation on characteristics was sought concerning age, education, industry of work, place of residence, and symptoms of emotional exhaustion and depression (measured with MBI-GS and SCL-6 respectively), with the aim of gaining a varied sample in accordance with maximum variation purposeful sampling. Potential participants were contacted via e-mail and telephone with information about the study and asked about participating in an interview. Recruitment of participants continued until the dataset’s information power was deemed adequate [43]. A total of 25 women were contacted. Among them, 15 participated, 8 declined, and 2 were excluded due to age. Table 1 summarises the characteristics of the participants.

**Table 1:** Summary of characteristics of participants

Participant characteristic	
Age in years (mean (min.-max.))	30 (25–35)
Education (n)	
Unknown	1
Secondary school	3
University	11
Industry of work^a^ (n)	
Female dominated e.g. health and social care	6
Gender integrated e.g. knowledge intensive services	2
Male dominated e.g. machinery operations	7
Place of residence (part of Sweden) (n)	
Northern part of Sweden	4
Metropolitan regions (3 regions of Sweden)	6
Other parts of Sweden	5
Symptoms of emotional exhaustion or depression^b^ (n)	
Yes	8
No	7

^a^Härenstam & Nyberg (2021) [44]

^b^The variables were depression, anxiety, and stress. Anxiety was most frequent

### Data collection

Data were collected through 15 individual semi-structured interviews with women between 25 and 35 years old, working in a variety of industries, living in different parts of the country, some of whom were experiencing symptoms of emotional exhaustion and depression. The majority of participants had a university education. An interview guide was used during the interviews, the areas of the guide are outlined in Table 2. A prompt in the form of ladder scale illustrating social status position was used as a starting point for the conversation. The wording of the interview question was based on an SSS scale question, similar to that used by Adler et al. [22] but simplified. References to money, education, and jobs were omitted to allow the participants freedom to consider the subject without being influenced by the question itself. For the same reason, the interview question did not specify a comparison group, leaving it open for the participants to reflect on and discuss during the interview.

The order of the questions was adapted during each interview in order to have a natural flow in the conversation. To obtain as rich descriptions as possible, probes and follow up questions were also used, as were interpreting questions [45]. The interview guide was tested in three pilot interviews with persons similar to the intended participants [45], by three of the authors (ÅA, ID, and YL). During the piloting process, the prompt (in the form of a ladder scale) was introduced, and some changes were made to the way the questions were phrased. The 15 study interviews were carried out during June 2023–Sept 2023 by the last author (ID). The interviews were conducted via the e-meeting service Zoom, using end-to-end encryption, and lasted between 21 and 44 min (average 31 min). The interviews were audio-recorded and transcribed verbatim.

**Table 2:** Main areas and questions of the interview guide, and typical follow-up questions

**Subjective social status**
[*Show picture of the ladder*] The ladder shows peoples’ position in a group or society. At the top of the ladder is the highest social status. At the bottom of the ladder is the lowest social status. Where on the ladder would you place yourself today? Please elaborate what makes you place yourself right there. What would have made you place yourself lower/higher?
In what ways does social status matter in your life?
**Social status and mental health**
Different aspects of social status—how are you affected?
**Social status and social media**
In what ways are social status and social media connected?
**Social media and mental health**
In what ways does social media affect how you feel?
**Follow-up questions** depending on individual answers: Can you tell me a bit more? Can you tell me about a situation when this happens? Can you give an example? Can you elaborate…? When you say…, what does that mean to you?

### Data analysis

Analysis was performed using thematic analysis [46], a method used for identifying patterns of meaning, or themes, across data. The analysis followed the six phases described by Braun and Clarke [46]. An inductive approach was used, and coding was performed at a semantic level. Three of the authors (ÅA, ID, and YL) performed the main analysis together, working on coding and initial themes and holding recurrent discussions about tentative results. Coding of the data were discussed until consensus was reached in the analysis group. The analysis went back and forth between phases, as needed. Regular meetings were held with the whole project group, where the analysis process and tentative results were discussed to ensure consensus. The authors have different professional backgrounds, such as nursing, public health, psychology, physiotherapy, and political science. The authors’ expertise includes qualitative research methods and research areas such as working life, vocational rehabilitation, stress research, behavioural medicine, and health services research. All authors were women. The analytic process took place over several months. NVivo 14 software was used in data management.

### Ethical considerations

The project received ethical approval from the Swedish Ethical Review Authority (dnr: 2022-00417-01). Participants were given information, both in written form and orally, about the purpose of the study, the contents of the interviews, confidentiality, and that participation was voluntary, and that participants could withdraw from the study at any time. Informed consent was obtained from all participants included in the study.

## Results

The thematic analysis identified several determinants and processes describing how the participants perceived social status, and a number of health consequences and processes describing its impact, resulting in two themes: *The qualities and processes of subjective social status* and *the influence of subjective social status on health and well-being*, each consisting of two subthemes.

### The qualities and processes of subjective social status

Social status was perceived as being dependent on several aspects and shaped by an ongoing interactive process. Two subthemes summarises these findings: *material and personal resources* and *interpersonal and contextual interplay*.

#### Material and personal resources

A range of aspects were seen as contributing to social status, including traditional measures of socioeconomic status, as well as background characteristics and aspects related to life values and quality of life.

Traditional socioeconomic measures, such as high levels of education, a prestigious occupation, and high income, were seen as important aspects that substantially contributed to increasing subjective social status. Being successful and having a secure career were seen as enhancing status, as was having good working conditions and longer vacation time. Aspects with potential negative effect on social status were not being as far ahead career-wise as others, being unemployed, let go from a job, or on sick leave. In addition, having a job that was considered not associated with high achievers, or perceived to involve work that not many people were willing to perform, affected social status negatively. Alongside income, the participants spoke more generally about having good personal finances as a determinant of social status, with financial problems such as receiving claims from the Enforcement Authority (*Kronofogden)*, considered as negatively affecting social status. The traditional socioeconomic measures were described as interconnected; with education, for example, described as a prerequisite to achieve a more prestigious job or a higher salary, and good personal finances considered the precondition to consumption and experiences; for instance, regarding area of living, type of housing, and vacations.

There were a number of non-influenceable background characteristics that were seen as affecting social status. Among such factors was age, with older age increasing status. The idea that higher age enhanced social status was generally seen as connected to life experience and knowledge, and when described in relation to work, connected to establishing oneself in the work role. Social background was seen as influencing social status, where a safe and stable upbringing with supportive parents, a rich social network, and financial security within the family were described as positive factors. Some participants born outside of Sweden or with parents born outside of Sweden spoke of this aspect of their background as a factor lowering social status. They were often reminded about being different; for instance, by being addressed in other languages than Swedish, leading to a sense that their identity was always in question. Being a woman was furthermore seen as contributing to lower social status by the participants. They described a dynamic where the more men there were in a group or in a workplace, the lower their social status became, as women. Participants also spoke about men having higher salaries, and not being as affected career-wise if they had children. The participants perceived there to be a different set of rules for women and for men, where women were judged more harshly than men.

*“…if you have too much confidence*,* then you are… they are self-centred or something*,* but if you have too little confidence then it is also… well then it is unfortunate*,* because you are supposed to be*,* you are supposed to be an independent and strong woman*,* but not too independent and strong…” (Interview 9).*

Moreover, a social network, close relationships, and a rich social life, were seen as important for subjective social status. This involved having a large social network, living close to where things are happening, going out and participating in events, and having a good grasp of social codes. Having both close friends and many friends were seen to increase social status, as was having a family, defined as a partner and children. Furthermore, feeling free to choose how to spend your time was valuable in and of itself, whether this was with family or friends in spontaneous social activities. Being in control of your life—having the possibility to realise your ambitions—was also important. Being involved in associations such as sports clubs was considered to increase social status, as was the type of interest, with, for example, physical exercise and golf considered to be high-status activities.

Another important aspect contributing to social status was competence and trustworthiness, which were not necessarily connected to professional life. Knowledge and proficiency were positive for social status; for instance, regarding leadership, rules, and regulations, or specialist knowledge in a field. Additionally, having others listen to and value what you say, as well as earning their trust, also enhanced social status. Not acting seriously or in a socially expected manner was seen as lowering status. Moreover, ambition—or being dedicated and developing as a person—was considered to increase status, while not having any plans or visions, or being generally passive, lowered social status.

Finally, good health, and specifically good mental health, was seen as an aspect affecting social status positively. Appearance and fulfilling beauty ideals were also seen as important, in the sense of looking good or not gaining weight, but also in the sense of height, where being short was considered to decrease social status.

#### Interpersonal and contextual interplay

The establishment of social status was perceived by the participants as a dynamic and interactive process encompassing different aspects. Social status was viewed as dependent on external influences; something that is created in interaction with others. It is concerned with what matters according to society and what others value and strive for. It was also seen as being context-dependent, with the possibility of having higher social status in some contexts and lower social status in others. The context could, for example, be society in general, but also the workplace, or social arenas.

*“Because I guess that social status is something that…one person can’t decide that*,* rather it is something societal that is decided by many…” (Interview 11).*

*“… I think that I have a different position there based on…there it might be a lot about personality and what*,* what I do…so…well*,* I am a member of an athletic club and am involved and active there and there I sort of know that they see me in a different way than maybe some colleagues do…” (Interview 7).*

Participants described comparing themselves to others when deciding where to position themselves status-wise. This could be both upwards and downwards; for instance, determining that others had more friends or came from a more privileged background, or that you could afford things that your parents could not afford at the same age. This comparison was also present on social media, where participants could place what they saw in relation to their own lives, even though there was a consciousness that what one sees on social media is not necessarily the whole picture.

Determinants of social status were perceived to be balanced against each other. For instance, type of work could lower social status but be balanced by a high salary, poorer background conditions could be compensated by striving for higher education, or high social skills could matter more than the money you make.

*“I mean*,* it doesn’t matter if someone is very rich and has a very successful job and so on*,* if you are very disagreeable or when…it’s still in the meeting between people it becomes real somehow…they can have a higher social status in all parameters but in meeting with other people perhaps their status is lowered a lot if they don’t understand codes or how other people feel and such…” (Interview 8).*

### The influence of subjective social status on health and well-being

Initially, during the interviews, social status and social media were described as not having a significant impact on the participants' well-being. However, after reflecting on the interview questions the respondents elaborated on their views and described in detail how they were affected in regard to their health and well-being. Two subthemes were identified describing these results: *being judged by oneself and others* and *strategies to reduce negative impact.*

#### Being judged by oneself and others

The participants described emotional responses related to subjective social status. These were, for instance, guilt or shame if social status was perceived to have decreased due to sick leave and being out of work, or sadness and anger if one felt they had been written off by others in advance. Having to handle social status matters by either trying to ignore them or proving things to others felt draining, and pressure and stress related to always having to have the right things and do the right things were also described.

*“Yes*,* but it could also be in situations where it feels*,* again where it feels important to make a good impression and where it feels like I won’t be able to do that if…because I can make a really good impression and then they find out I’m not a lawyer…(laugh)*,* well*,* like that.” (Interview 7)*.

Some participants stated that what they saw on social media could inspire them in positive ways, regarding, for instance, physical activity. However, participants also described being continuously fed input from social media about how one is supposed to be, which led to a shift in their worldview based on those images. Although they realised that social media affected them in this way, they found it difficult to protect themselves from its influence. The perception became that the normal state in life is happiness and success, and that their view of their own appearance was affected. This resulted in a sense of pressure to share pictures of their lives on social media and to curate what they displayed there.

*”…but on the other hand*,* I am being fed a certain type of thought about how you are expected to be and how you are considered successful and how…and that can really affect me…and even just*,* it doesn’t have to be sort of more official people*,* it can also be that my acquaintances… everyone chooses to just post things when it is very good. And that makes you*,* or I feel that I am affected*,* and I know that everyone around me also feels it*,* that you are very affected by people only posting everything good*,* and then you feel maybe that you yourself don’t have it as good*,* because no one has it as good as on social media.” (Interview 11)*.

Having a lower social status was perceived as leading to others looking down on you, thinking you have nothing to show for yourself, or feeling that you don’t dare to be yourself due to fear of how others will perceive you. High social status, on the other hand, leads to others looking up to you, taking you seriously, and seeing you as correct and orderly. This could give a feeling of being seen as an equal and getting better treatment from others. The participants also relayed the view that social status affected their perception of being included or excluded in different situations; for example, feelings of connection or community, or, in contrast feeling left out or alone. Not fitting in because of your background or feeling left out for being unemployed were some of the examples given. Participants described that social status affects how others view and treat you.

*“…I trust myself that I know something and that I am capable*,* that what I know is right*,* that I can stand behind what I know…and that makes the social part lift too*,* because you are more sure of yourself*,* and then…then it is also socially*,* that you are more accepted and welcome and involved.” (Interview 12).*

#### Strategies to reduce negative impact

Participants’ own approaches and attitudes towards subjective social status differed and could regulate its impact. For example, some participants recounted being disinterested in social status or being content with their current status level. Social status was reported as not being very important, something you didn’t care much about, or something you simply didn’t think about.

Participants described being content with what they had achieved and what they had in life; that it was good to be somewhere in the middle on the social status ladder, since both higher up and lower down, you risk being in a vulnerable position. It was seen as important not to decrease in social status, but also good to not have the pressure and stress of having a very high social status, as this meant that one had a lot to live up to. The approach of being disinterested or content could also be seen in regard to social media, where some participants expressed that they didn’t chase status on social media or cared overmuch about the image of themselves there.

*“For me it’s not important to be high up. I don’t think so. I don’t strive to be at maybe a nine or a ten or something*,* no I’m rather content where I am. I mean I have my friends; I know that I*,* that they are close*,* and they appreciate me for who I am. And the same thing with those who*,* that is*,* my family and so that I have around me*,* it’s like…yes. I do feel that I have what I need*,* and I don’t have to strive for higher social status to be satisfied with my life.” (Interview 6).*

Regarding social media and approaches to social status, participants described an awareness of distortion, where what is shown on social media is a polished front that is arranged and fixed and not as perfect as it seems; that what you see is not reality but has a positivity bias. Participants found this to also concern themselves, that they felt it was important to portray themselves in accordance with different ideals, and with that came reflections about what they did and what they truly wanted to display on social media. However, there was also the opposite, where the participants described trying to be honest on social media, whether things are going well or not, not feeling like they need to put up a facade, or wanting to show a more realistic picture.

*“…no*,* but it feels like before you did things more because you wanted to do them in the moment maybe*,* but now a lot is to show you know life on social media and you only show the good and positive sides*,* […] I usually think about it sort of like I have two lives*,* that there is one life that takes place there*,* but it doesn’t hold much truth*,* and there is one life that takes place here. And unfortunately*,* you are pretty stupid and when you look at others you think it is their reality*,* but it isn’t*,* it’s only selected parts…” (Interview 4)*.

There was also a perception that comparison with others can affect you negatively, if you are currently already in a more vulnerable position; for instance, if being out of work and seeing friends and acquaintances progressing their careers, or feeling left out when watching others’ social lives on social media.

Participants described ways in which they handled issues related to subjective social status through active management. For example, they described a need to assert or prove themselves as being smart or knowledgeable, and to gain acknowledgement for achievements.

*“I can feel that I need to…assert myself*,* perhaps…maybe bring evidence that I am actually smart*,* things like that. I can maybe think it’s hard if*,* if I make some mistake in another way*,* depending on who notices the mistake.” (Interview 7).*

Some participants described this as overcompensating or being unaccommodating in relation to demands from co-workers after initially feeling like they needed to be accommodating, or as not always acting as congenially as they might want to in situations where they had stood their ground regarding a disagreement. The participants also described how they consciously regulated their social media use to protect themselves from negative influence. For example, they limited the content and refrained from following accounts that they felt affected them negatively; for example, accounts with very physically fit women, and followed accounts that had more of the values that made them feel good. One of the participants described it as trying to steer your social bubble to where you want it to be.

## Discussion

The aim of this study was to explore the perceptions of young working women in Sweden regarding social status and how it relates to their health and well-being. Two main themes were identified. The first theme *the qualities and processes of subjective social status*, comprised the subthemes *material and personal resources* and *interpersonal and contextual interplay.* The second theme *the influence of subjective social status on health and well-being* comprised the subthemes *being judged by oneself and others* and *strategies to reduce negative impact*. The discussion is structured according to the themes and subthemes below.

### The qualities and processes of subjective social status

The theme *the qualities and processes of subjective social status* is discussed according to the subthemes *material and personal resources* and *interpersonal and contextual interplay.*

#### Material and personal resources

Several different aspects were perceived to affect social status, among them the traditional socioeconomic factors of education, occupation, and income, but also other factors such as social life and appearance. These results are consistent with previous quantitative [11] as well as qualitative [26] studies that showed several other factors were important for social status in addition to the traditional socioeconomic factors. For example, the participants expressed that being a woman affected social status negatively, and that as a woman, one is subject to a different set of rules compared to men. This can be noted also in earlier studies, reporting that it seems to take more for a woman to gain status than for a man [11], and that there are different conditions for girls and boys when studying social status in school settings [26, 27]. The sense of having to do more, but still not be ‘too much’ is evident also in the research by Hiltunen [26], where girls described having to balance between being too noticeable or not noticeable enough. Even though the results are in accordance with other research, it is noteworthy that these perceptions of differences are still apparent among young working women in Sweden, a country scoring high in international comparisons on equality [47]. Another background characteristic perceived to lower one’s social status was being born outside of Sweden or having parents born abroad. This is also in line with previous studies, showing discrimination against people on the basis of ethnicity [48, 49]. Our results indicate experience of such discrimination is also present among our sample of young, mainly well-educated women in Sweden; a country with a history of ambitious integration policies. The participants also described how different aspects can be balanced against each other to move you up or down the social ladder. These results align with a cognitive averaging principle, which suggests that individuals use an average of relevant aspects to arrive at their SSS [7–9]. The cognitive averaging principle can also relate to time, as seen in the participants’ reference to certain background factors in relation to social status. Future prospects were not as apparent, which might be due to the participants having already studied and started their careers.

Of least importance in relation to social status in a previous quantitative study [11] were factors like interest and engagement in politics, the environment and fine art, which also seems to be in line with the results of the present study. These aspects were not brought up in the interviews, except for being involved in associations such as sports clubs. Surprisingly, engagement in sustainability and environmental questions was not present in the results, even though these issues are prevalent among interests of young people and especially young women today. Earlier research has shown that women worry more about climate change than men [50]. The reasons for this not appearing in the results might be that the participants did not connect it to social status, or that the sample did not include women under 25, and these issues might be more prevalent in the younger end of the age interval. However, it was not an aspect raised among adolescents in the earlier qualitative study either [26], which may lend weight to the conclusion that this is not an aspect young women associate with social status.

#### Interpersonal and contextual interplay

Processes related to the establishment of social status included external influences, context-dependence, comparison, and balancing aspects. This description of the establishment of social status is very much in line with definitions of the concept such as those described by Blader and Chen [5], indicating that the participants were quite aware of the concept, even though they initially stated it was not something they thought much about, or something they saw as important. Being aware of social status and how it is established can be viewed as an expected result, since this has been found to be evident even among adolescents in school environments [26, 27].

### The influence of subjective social status on health and well-being

This main theme is discussed according to the subthemes *being judged by oneself and others* and *strategies to reduce negative impact*.

#### Being judged by oneself and others

The results included emotional responses related to social status. Many of these can be described as negative, e.g. guilt, shame, sadness, anger, feeling drained or stressed, feeling looked down upon, and that you can’t be yourself. There were also positive responses, such as feeling motivated, being seen as an equal, and being looked up to. The fact that the emotional responses can be both negative and positive aligns with findings from a study on adolescents, were emotions like jealousy, shame, motivation, and thankfulness were present [28], while another only presented negative reactions such as worry, anxiety, and feeling inadequate [26]. Among girls in school-settings there was also apparent the importance of inclusion and being part of the group [26], something also visible among the young working women, who described that social status affected their perception of being included or excluded.

The results included an awareness of distortion regarding what one sees on social media, but also the feeling that the constant input could shift how one views things. This differs in some respects from the results in the study by Solomon [40], where the perception that one did not identify with images or found them to be unrealistic counteracted negative feelings. Results are, however, similar in that exposure to social media content in general led to participants questioning their personal and social status, and could lead to body image issues. This is also in line with adolescents finding media input to affect them and creating ‘templates’ for how you are supposed to be and what you are supposed to have [26].

As mentioned above, participants described a feeling of being fed a continuous input in social media which could shift one’s worldview and affect how individuals view themselves. In social comparison theory [33], the increased importance of an opinion or ability increases the pressure to decrease differences in that opinion or ability. If it is not possible to decrease the difference, and an individual deems themselves to be lower than others, that can lead to feeling inadequate and to feelings of failure. The theory also states that one’s range of comparison should narrow and exclude those who are very different; however, in the present study it was described by the participants as difficult to completely ward against the constant flow of input they experienced.

#### Strategies to reduce negative impact

In response to input on social media, participants used strategies to ameliorate the effects, such as trying to regulate their social media use to experience less unwanted input. This can be seen as being in line with social comparison theory [33], which states that people tend to move in and out of groups in order to have opinions and abilities near their own as a point of comparison, while also lowering the risk of forming a negative evaluation of oneself. This part of the theory can also be put into relationship to participants describing being content with where they ranked themselves, status-wise. As stated earlier, both in our results and in previous studies focusing on adolescents [26, 27] there was an awareness of social status and how it is established, along with a sense that women and girls have a different set of rules than men and boys. In the present study, some participants described strategies were they actively went against the expected, where women are accommodating and act congenially, to reduce negative impact on themselves. Another strategy found in the results was to be content with what you have and where you are. Participants expressed relief about being somewhere in the middle of the status ladder as it would be stressful to be both lower and higher. This strategy, and the reasoning for it, can be found also among adolescents expressing being satisfied to have a position that is less demanding [27].

### Strengths and limitations

One strength of the present study is its focus on the group of young working women, a group not previously extensively investigated. The sampling strategy was successful in including women differing in most of the intended variables: age, education, industry of work, place of residence, and symptoms of emotional exhaustion and depression. Furthermore, the use of the social status ladder scale as a prompt in the interviews worked well to narrow the focus in conversations to the participants’ themselves, rather than risking the focus being too general.

To ensure trustworthiness of the results, several strategies were used [41]. Credibility was sought by using analyst triangulation in the form of three of the research team members collaborating in the analysis process, as well as two of the research team members performing ‘critical friend’ review. Another strategy for ensuring credibility was pilot testing of the interview guide with individuals as similar to the participants as possible. Dependability was addressed through clearly documenting the research process, and confirmability through linking findings and interpretations to the data by using quotations to illuminate findings. To facilitate assessment of transferability, the research process has been described in as much detail as possible [41].

Some limitations of the study can be noted. The sample did not include any participants younger than 25 years, which might have affected the results; the experiences and perceptions of 20-year-olds might differ significantly from women in their mid-twenties to mid-thirties. Thus, the results may be transferable to similar samples concerning background variables and in similar settings. Another limitation of the study is the sample size, which was small, however the information power [43] of the dataset was deemed adequate. Furthermore, the social status ladder scale used as a prompt was kept open to the participants’ interpretation as to the comparison group, an aspect which has been shown to have relevance for where an individual places themselves on such a scale. It is possible that the results could have differed if the question had focused only on one particular reference group, however the focus of the study was broad and part of the results is the participants’ views on the relevance of different arenas for social status.

### Policy implications and future research

In regard to implications for policy and practice, the results suggest that interpersonal processes linked to social status in general and on social media specifically can be significant stressors for young working women. This finding is an important message to employers aiming to create a health enhancing work environment, and to professionals meeting young working women with mental ill-health. Future qualitative research should focus on the establishment and impact of subjective social status in samples including women under 25 years of age. Other directions could be focusing on other countries or cultures, or looking at the question in relation to more specific comparison groups, such as the community, when using the ladder as a prompt. It would also be of interest to investigate whether the strategies to reduce negative impact also have an effect on how individuals place themselves on the ladder. Moreover, it is worth investigating how young men view these questions, since the issue of mental ill-health is present in this group as well. Future quantitative studies are needed to investigate the role of social media in the status processes, and also the relation to health and well-being.

## Conclusions

This study focused on the perceptions of young working women in Sweden concerning social status and its impact on their well-being. The results indicate that subjective social status is established through dynamic and interactive processes involving several aspects of importance, including, but not limited to, traditional socioeconomic measures. Notable results are that among these young working women, being a woman was—even today—perceived as something affecting social status negatively, as was being born outside of Sweden or having parents born outside of Sweden, indicating a need for continued efforts to strengthen equality and integration. Social status seems to be linked to well-being by influencing emotional distress, affecting one’s sense of community, and how others view and treat you. A contribution to the SSS literature is the results pertaining to the participants handling of the impact of social status, which involved processes such as the individual’s approach to social status, and active management of negative influence from, e.g., social media.

## Data Availability

The datasets generated and analysed in this study are not publicly available due to the principle of confidentiality. For any information regarding the transcripts of the interviews or the data analysis, the corresponding author may be of assistance.

## Abbreviations

SSS: Subjective Social Status
